# Antidepressant-like effects of acupuncture involved the ERK signaling pathway in rats

**DOI:** 10.1186/s12906-016-1356-x

**Published:** 2016-09-29

**Authors:** Xuhui Zhang, Yingzhou Song, Tuya Bao, Miao Yu, Mingmin Xu, Yu Guo, Yu Wang, Chuntao Zhang, Bingcong Zhao

**Affiliations:** 1 Beijing University of Chinese Medicine, Beijing, Beijing 100029 China; 2 Shanxi University of Chinese Medicine, Xianyang, Shanxi 712046 China

**Keywords:** Acupuncture, Depression, Extracellular signal-regulated kinase (ERK), Chronic unpredictable mild stress (CUMS), Neurogenesis

## Abstract

**Background:**

The extracellular signal-regulated kinase (ERK) signaling pathway is considered to be associated with the pathogenesis and treatment of depression. Acupuncture has been demonstrated to ameliorate depression-related behavior and promote neurogenesis. In this study, we explored the role of the ERK signaling pathway in the antidepressant-like effects of acupuncture in rats exposed to chronic unpredictable mild stress (CUMS).

**Methods:**

Eighty male Sprague–Dawley rats were randomly divided into eight groups: control group, model group, model + Acupuncture group (Acu group), model + fluoxetine group (FLX group), model + DMSO group (DMSO group), model + PD98059 group (PD group), model + Acupuncture + PD98059 group (Acu + PD group) and model + fluoxetine + PD98059 group (FLX + PD group). Except for the control group, all rats were subjected to 3 weeks of CUMS protocols to induce depression. Acupuncture was carried out for 10 min at acupoints of Baihui (GV-20) and Yintang (GV-29) each day during the experimental procedure. The ERK signaling pathway was inhibited using PD98059 through intracerebroventricular injection. The depression-like behaviors were evaluated using the sucrose intake and open-field tests. The protein levels of ERK1/2, phosphor (p)-ERK1/2, cAMP response element-binding protein (CREB), p-CREB and brain-derived neurotrophic factor (BDNF) in the hippocampus were examined using western blot.

**Results:**

Acupuncture ameliorated the depression-like behaviors and dysfunction of the ERK signaling pathway in the hippocampus of CUMS rats. PD98059 pretreatment inhibited the improvements brought about by acupuncture on the ERK signaling pathway.

**Conclusions:**

Taken together, our results indicated that acupuncture had a significant antidepressant-like effect on CUMS-induced depression model rats, and the ERK signaling pathway was implicated in this effect.

## Background

Depression is a common mental disorder and a leading cause of disability throughout the world [1, 2]. Antidepressants remain the main clinical treatment for depression [3, 4]; however, antidepressants cannot completely meet the needs of depression patients [5]. Acupuncture, an important part of traditional Chinese medicine, has been shown promising effects in alleviating the progression of depression [6, 7]. However, the underlying mechanism is poorly understood.

**Fig. 1:**
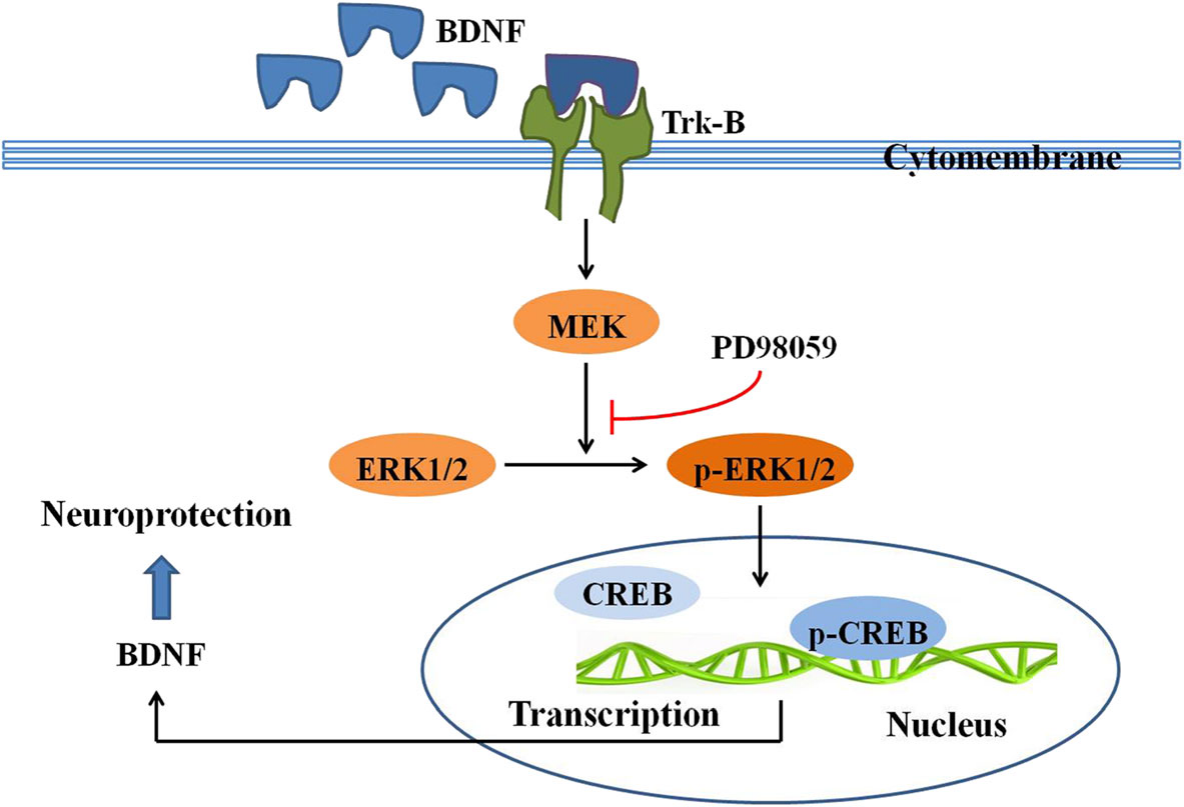
The ERK signaling pathway. BDNF combines with its specific receptor Trk-B and the compound object can activate the Ras-dependent cascade. The Ras-dependent cascade is Ras-Raf-MEK-ERK signaling pathway. Phosphorylated ERK1/2 (p-ERK1/2) can further phosphorylate CREB and promote the transcription of BDNF mRNA. PD98059 prevents the ERK1/2 from being phosphorylated. Activating reactions were depicted by an *arrow* with a *black* color and the inhibiting effect of PD98059 was depicted by a *line* with a *red* color. The proteins involved in the present study were marked by a *rectangle box* with a *black* color. *BDNF* brain-derived neurotrophic factor, *CREB* cAMP response element-binding protein, *ERK1/2* extracellular signal-regulated kinase 1/2, *MEK* ERK1/2 kinase, *p-CREB* phosphor-CREB, *p-ERK1/2* phosphor-ERK 1/2, *Trk-B* tyrosine receptor kinase B

In the last few years, studies have found that a decline in neurogenesis might be an etiological factor in depression [8, 9]. Brain-derived neurotrophic factor (BDNF) has been shown to mediate neurogenesis and synaptic plasticity, which is implicated in depression pathogenesis [10]. The extracellular signal-regulated kinase (ERK) 1/2, a downstream target of BDNF, is activated by the binding of BDNF to tyrosine kinase receptor-B (Trk-B) via the Ras-dependent cascade, including phosphorylation (p) of transcription factors such as cAMP response element-binding protein (CREB) [11] (Fig. 1). Some studies have proved that a decrease in ERK1/2 expression leads to depression-like behaviors in model rats [12–14]. Antidepressants can alleviate the symptoms of depression by increasing ERK1/2 [15] and p-ERK1/2 expression [16–19].

Recent studies showed that acupuncture produced the neuroprotective effects in many neurological disorders [20], including up regulating the gene and protein expression of BDNF in hippocampus [21, 22], reducing neural apoptosis and promoting adult neuron neurogenesis [23, 24]. Additionally, our previous study found that acupuncture activated the proteins expression of phosphor (p)-ERK1/2 and p-CREB in the hippocampus and prefrontal cortex in depression model rats [25]. Therefore, we had a hypothesis that the antidepressant-like effect of acupuncture might via modulation of the ERK signaling pathway [25, 26].

The aim of the present study was to investigate the regulation of acupuncture on the depressive-like behaviors and the ERK signaling pathway as well as BDNF protein expression in chronic unpredictable mild stress (CUMS)-induced depression model rats. We used PD98059 pretreatment to inhibit the activity of p-ERK1/2; then, we evaluated the behavioral activities by the sucrose intake and open field tests, as well as the protein levels of ERK1/2, p-ERK1/2, CREB, p-CREB and BDNF in the hippocampus by western blot analysis.

## Methods

### Animals

Eighty male Sprague–Dawley rats aged 8 weeks old and weighing 180~200 g each were obtained from Vital River Laboratory Animal Technology Co. Ltd, China (license number SCXK [Jing] 2012-0001). The experimental procedures were conducted in compliance with the *Guidance Suggestions for the Care and Use of Laboratory Animal*, issued by the Ministry of Science and Technology of China [27] and received approval from the Animal Ethics Committee of Beijing University of Chinese Medicine (permission No. Kj-dw-18-20140923-01).

### Overall research design

**Fig. 2:**
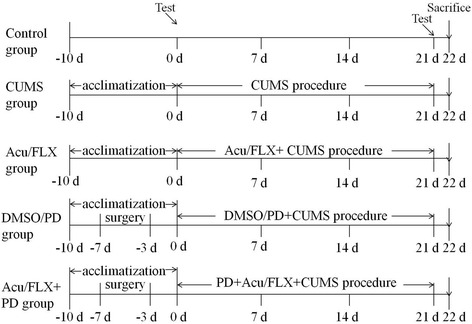
Overall research design

All animals were fed ad libitum, and housed at 23–25 °C on a 12 h light/dark cycle (lights on between 7:00 A.M. and 7:00 P.M.). They were randomly divided into eight groups (*n* = 10 per group) as follows: (1) The control rats were not subjected to any stress except general handling for 3 weeks. (2) The CUMS group rats were exposed to CUMS for 3 weeks. (3) The Acu group rats received acupuncture stimulation and CUMS for 3 weeks. (4) The FLX group rats received fluoxetine treatment and CUMS for 3 weeks. (5) The DMSO group or (6) PD98059 group (PD group) rats were exposed to CUMS and intracerebroventricular injection of either dimethylsulfoxide (DMSO) or PD98059, respectively, for 3 weeks. Then, the groups in which PD98059 was administered were treated in the same manner as the PD group except (7) acupuncture (Acu + PD group) or (8) fluoxetine (FLX + PD group) was also administered. One rat in the Acu + PD group died during stereotactic surgery; thus, 79 rats were involved in the final analysis. The overall research design is shown in Fig. 2.

Fluoxetine is a classic antidepressant that belongs to selective serotonin reuptake inhibitor medication. The recent study demonstrated that fluoxetine-induced the increase in BDNF protein level was accompanied by activating the ERK signaling pathway [28]. So we chose fluoxetine as the positive medication to evaluate the effect of acupuncture on the ERK signaling pathway. PD98059 is dissolved in DMSO to meet the concentration that can inhibit the ERK signaling pathway [29, 30]. DMSO is characteristic of anti-inflammation, analgesic and promoting blood circulation. Besides, the placement of cannula is an invasive surgery. So we set the DMSO group and compared it with the CUMS and PD groups respectively to eliminate the interferences of DMSO and the surgery to the behavior tests and the indexes.

### Chronic unpredictable mild stress procedure

The chronic unpredictable mild stress (CUMS) model was modified from the methods previously described [31] and has been validate as one of the most relevant models of depression. Except for the control group, all rats were exposed to CUMS after 10 d of acclimatization under the housing conditions. Seven different stressors were used to model a state of depression as follows: food deprivation (24 h), water deprivation (24 h), wet bedding (24 h), overnight illumination (12 h), using a restraining device (2 h), shaking the cage on a rocking bed (30 min), and clamping the middle of tail with a binder clip (3 min). The CUMS procedure was carried out for 3 weeks and a different stressor was administered randomly each day.

### Intracerebroventricular injection

Rats were anesthetized with 350 mg/kg 10 % chloral hydrate i.p. and placed in a rat brain stereotactic frame (RWD, Shenzhen, China) with the incisor bar positioned 4 mm below the interaural zero. A burr hole (0.9 mm posterior to the bregma; 1.5 mm lateral to the midline) was drilled through the parietal bone and a stainless-steel guide cannula (RWD; 0.58 mm outside diameter [OD], 0.38 mm inside diameter [ID], 3.5 mm under the dura [L]) was positioned in the lateral ventricle and secured with screws and dental acrylic onto the skull, serving as a guide for the accurate insertion of a internal cannula (RWD; 0.36 mm OD, 0.20 mm ID, aligning to the tip of the guide cannula). A cap (RWD; 0.36 mm diameter, aligning to the tip of the guide cannula) was always placed in the guide cannula and removed during the injection to prevent clogging or infection in the brain tissue. Reflux of cerebrospinal fluid from the guide cannula verified the correct placement of the intracerebroventricular cannula. The rats were given 3 d to recover after the surgery. After recovery, either 5 μL PD98059 (100 μM, dissolved in DMSO) or 5 μL DMSO was delivered by micro-injection with a pressure equalizer tube connected to the internal cannula. After injection, the internal cannula was left in the guide cannula for 1 min to ensure proper delivery.

### Acupuncture and drug treatment

Acupuncture was performed at the acupoints of Baihui (GV-20) and Yintang (GV-29) in Acu group and Acu + PD group each day during the experimental procedure. Sterilized disposable stainless steel needles of 0.3 mm diameter were inserted as deep as 2–3 mm. GV-20 is located above the apex auriculate, on the midline of the head. GV-29 is located at the midpoint of the two eyes. The acupuncture treatment was manually delivered by twisting the acupuncture needles at a frequency of twice per second for 1 min, and then the needles were retained for 10 min [25]. The FLX group and FLX + PD group were treated with 2.5 mg kg^−1^ d^−1^ fluoxetine (intragastric administration) each day during the experimental procedure.

### Behavior tests

The sucrose intake test (SIT) is used as a measure of anhedonia [32]. Before the first SIT, the rats were habituated to consume 1 % sucrose solution for 24 h without any water and then deprived of water for 24 h. The rats were then given a 2-h window for SIT between 12:00 and 14:00 h. The amount of sucrose consumed was measured by comparing the bottle weight before and after the 2-h window. The SIT was performed at beginning (0 d) and at end (21 d) of the experiment.

The open-field test (OFT) is commonly used to measure general locomotor activity and willingness to explore in rodents [33]. In the OFT, the rat was gently placed at the center of a square arena, which was a four-sided 80 × 80 × 40 cm^3^ box with the floor and walls painted black. The arena was divided into 16 × 16 equal squares that had been drawn on the floor. Each rat was allowed to freely explore the arena for 5 min. The activity of the rat was recorded by a video camera installed on top of the lateral high wall, similar to our previous experiment [34]. Two observers, blind to the experiment, counted the crossing number (defined as at least three paws in a square) and the rearing number (defined as the rat standing upright on its hind legs). After each animal was tested, the box was cleaned with a 10 % ethanol solution to remove any olfactory cues. The OFT was also performed at beginning (0 d) and at end (21 d) of the experiment.

### Western blotting analysis

Hippocampal tissue from rats was collected, immediately placed on dry ice, and stored at −80 °C until assay. Samples were homogenized in a standard lysis buffer supplemented with protease or phosphatase inhibitor cocktail, incubated on ice for 20 min, and centrifuged at 13,000 rpm for 20 min. The protein concentration was determined using a bicinchoninic acid (BCA) protein assay kit (Cwbio, Beijing, China). Proteins (24 μg) were separated on a 10 % sodium dodecyl sulfate-polyacrylamide gel electrophoresis gel (120 V, 60 min) and blotted (300 mA, 100 min) onto a polyvinylidene fluoride membrane. The membranes were blocked in 5 % bull serum albumin (BSA) tris-buffered saline plus Tween (BSA-TBST) for 1 h at room temperature (RT) and incubated at 4 °C overnight with the following primary antibodies: anti-ERK1/2 (catalogue NO.: 4965), anti-phosphor (p)-ERK1/2 (catalogue NO.: 4377), anti-response element binding protein (CREB) (catalogue NO.: 4820), anti-p-CREB (catalogue NO.: 9198), and anti-BDNF (catalogue NO.: ab46176) (1:1000 in 5 % BSA-TBST; Cell Signaling Technology, Danvers, MA, USA). Equal loading was confirmed using anti-β-actin (catalogue NO.: TA-09) (1:1000 in 5 % BSA-TBST; ZSGB-BIO, Beijing, China). Membranes were washed with TBST and incubated for 60 min at RT with horseradish peroxidase conjugated to goat anti-rabbit/mouse IgG (catalogue NO.: 111-035-003/115-035-003) (1:10,000 in 5 % BSA-TBST; Jackson ImmunoResearch Laboratories, Inc., West Grove, PA, USA). Immunoreactivity was visualized using enhanced chemiluminescence (Merck Millipore, USA).

### Statistical analysis

Data are presented as the mean ± SD. The comparisons of sucrose intake and western blot analysis between groups within the same time point were examined by one-way analysis of variance (ANOVA) method after the test of normal distribution and homogeneity of variance, followed by the LSD *post hoc* test. Since the crossing number and rearing number were not normally distributed, Kruskal-Wallis test was used, followed by the Mann–Whitney *U*-test. Statistical significance was defined as *P* < 0.05.

## Results

### Changes in sucrose intake

**Fig. 3:**
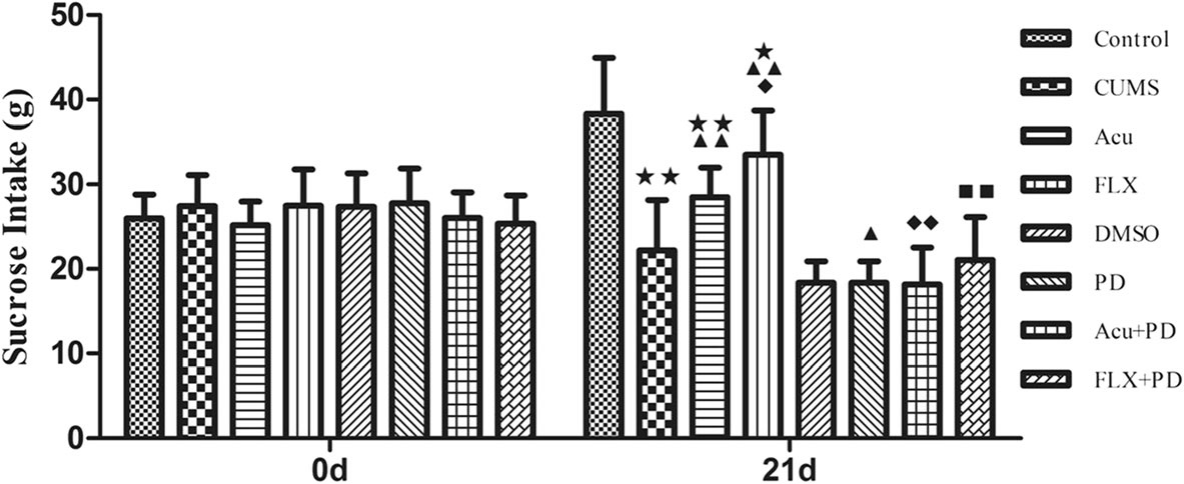
Sucrose intake at 0 d and 21 d. ^★^ *P* < 0.05, ^★★^ *P* < 0.01 versus control group; ^▲^ *P* < 0.05, ^▲▲^ *P* < 0.01 versus CUMS group; ^◆^ *P* < 0.05, ^◆◆^ *P* < 0.01 versus Acu group; ^■■^ *P* < 0.01 versus FLX group. (mean ± SD, *n* = 9–10)

At the beginning (0 d), there was no significant difference among groups [*F*_(7,71)_ = 0.862, *P* > 0.05]. At the end of the experiment (21 d), the sucrose intake was significantly different among groups [*F*_(7,71)_ = 26.302, *P* < 0.01] (Fig. 3 and Table 1). It was markedly reduced in the CUMS group compared to that in the control group (*P* < 0.01). Both acupuncture and fluoxetine treatment increased the sucrose intake (*P* < 0.01 for both), and fluoxetine treatment showed more effectiveness than acupuncture in the sucrose intake test (*P* < 0.05). However, they were still lower than those in the control group (*P*< 0.01 and *P* < 0.05, respectively). The difference between the CUMS and DMSO groups was not significant (*P* > 0.05), and the difference between the DMSO and PD groups was also not significant (*P* > 0.05); however, the sucrose intake in the PD group was lower than that in the CUMS group (*P* < 0.05). PD98059 pretreatment obviously inhibited the increase induced by acupuncture or fluoxetine (*P* < 0.01 for both).

**Table 1:** Sucrose intake at 0 d and 21 d

Group	Sucrose intake (g)	
	0 d	21 d
Control	25.96 ± 2.83	38.31 ± 6.63
CUMS	27.44 ± 3.68	22.17 ± 5.96^★★^
Acu	25.20 ± 2.77	28.49 ± 3.47^★★▲▲^
FLX	27.47 ± 4.29	33.51 ± 5.17^★▲▲^
DMSO	27.37 ± 3.95	18.35 ± 2.54
PD	27.77 ± 4.13	17.43 ± 3.96^▲^
Acu+PD	26.03 ± 3.02	18.15 ± 4.38^◆◆^
FLX+PD	25.37 ± 3.31	21.06 ± 5.10^■■^

^★^ *P* < 0.05, ^★★^ *P* < 0.01 vs. control group; ^▲^ *P* < 0.05, ^▲▲^ *P* < 0.01 vs. CUMS group; ^◆◆^ *P* < 0.01 vs. Acu group; ^■■^ *P* < 0.01 vs. FLX group. (mean ± SD, *n* = 9–10)

### Comparison of crossing and rearing numbers

**Fig. 4:**
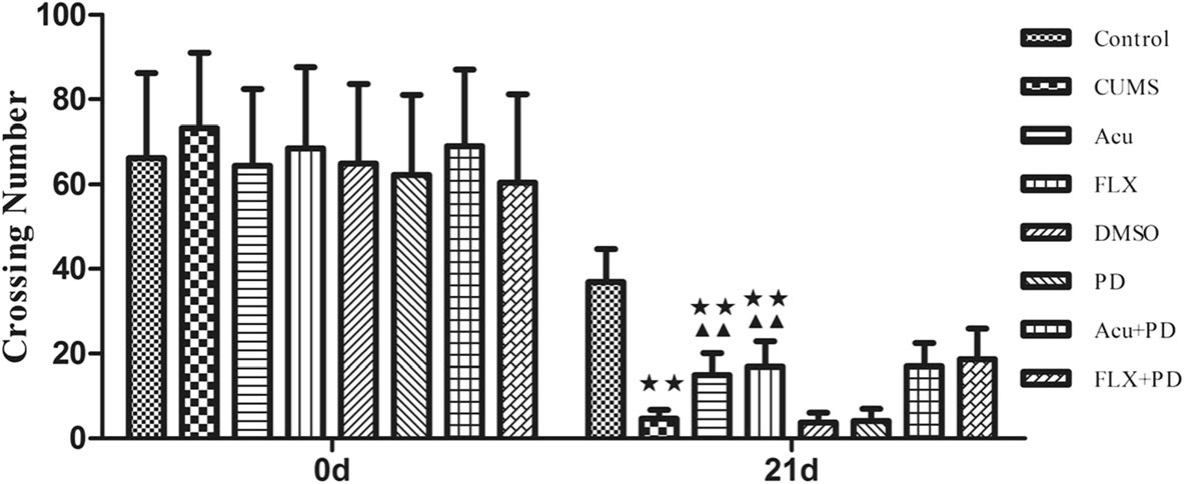
Crossing number at 0 d and 21 d. ^★★^ *P* < 0.01 versus control group; ^▲▲^ *P* < 0.01 versus CUMS group. (mean ± SD, *n* = 9–10)

**Fig. 5:**
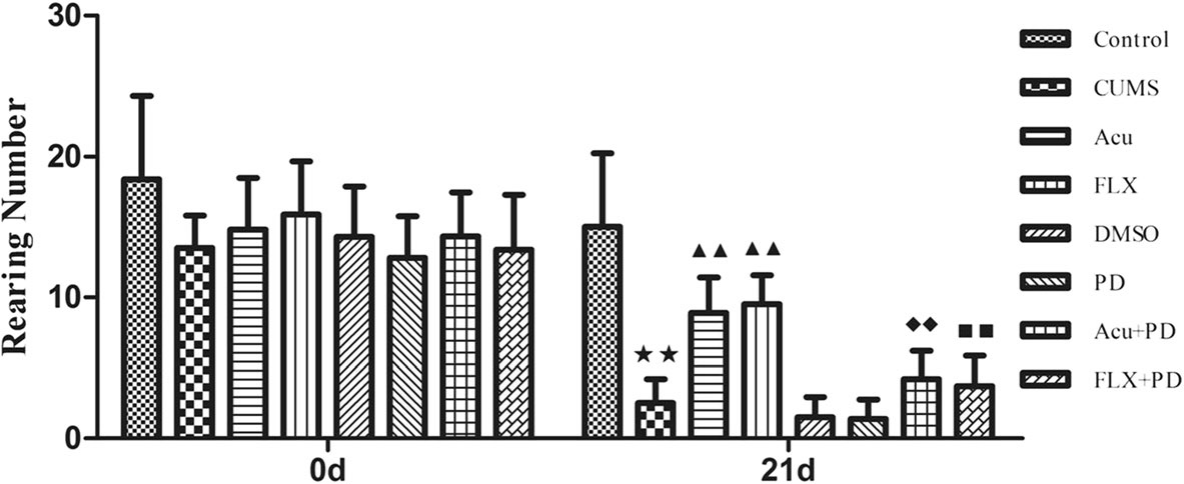
Rearing number at 0 d and 21 d. ^★★^ *P* < 0.01 versus control group; ^▲▲^ *P* < 0.01 versus CUMS group; ^◆◆^ *P* < 0.01 versus Acu group; ^■■^ *P* < 0.01 versus FLX group. (mean ± SD, *n* = 9–10)

There was no significant difference among groups in crossing [chi-square = 3.424, *P* > 0.05] (Fig. 4 and Table 2) and rearing numbers [chi-square = 8.831, *P* > 0.05] (Fig. 5 and Table 2) at 0 d. After the 21-d stress procedure, the crossing number (chi-square = 63.461, *P* < 0.01) (Fig. 4) and rearing number [chi-square = 61.099, *P* < 0.01] (Fig. 5) differed significantly among groups. Compared to the control group, CUMS rats showed a significant reduction in crossing and rearing numbers (*P* < 0.01 for both); however, both acupuncture treatment and fluoxetine treatment improved both crossing (*P* < 0.01 for both) and rearing numbers (*P* < 0.01 for both). There was no difference among the CUMS, DMSO and PD groups in crossing (*P* > 0.05) or rearing numbers (*P* > 0.05). The increase in rearing numbers induced by both acupuncture and fluoxetine was inhibited by PD98059 pretreatment (*P* < 0.01 for both); however, the increase in crossing numbers induced by acupuncture or fluoxetine was not affected by PD98059 pretreatment (*P* > 0.05 for both).

**Table 2:** Numbers of crossing and rearing at 0 d and 21 d

Group	Crossing number		Rearing number	
	0 d	21 d	0 d	21 d
Control	66.2 ± 20.0	36.9 ± 7.8	18.4 ± 5.9	15.0 ± 5.2
CUMS	73.3 ± 17.6	4.7 ± 2.1^★★^	13.5 ± 2.3	2.5 ± 1.7^★★^
Acu	64.4 ± 18.0	15.0 ± 5.1^★★▲▲^	14.8 ± 3.7	8.9 ± 2.5^▲▲^
FLX	68.5 ± 19.1	16.9 ± 6.0^★★▲▲^	15.9 ± 3.8	9.5 ± 2.1^▲▲^
DMSO	64.9 ± 18.6	3.8 ± 2.3	14.3 ± 3.6	1.5 ± 1.4
PD	62.2 ± 18.8	4.1 ± 2.9	12.8 ± 3.0	1.4 ± 1.3
Acu+PD	69.0 ± 18.0	17.0 ± 5.5	14.3 ± 3.1	4.2 ± 2.0^◆◆^
FLX+PD	60.4 ± 20.8	18.7 ± 7.2	13.4 ± 3.9	3.7 ± 2.2^■■^

^★★^ *P* < 0.01 vs. control group; ^▲▲^ *P* < 0.01 vs. CUMS group; ^◆◆^ *P* < 0.01 vs. Acu group; ^■■^ *P* < 0.01 vs. FLX group. (mean ± SD, *n* = 9–10)

### Western blot analysis of ERK1/2 and p-ERK1/2 in the hippocampus

**Fig. 6:**
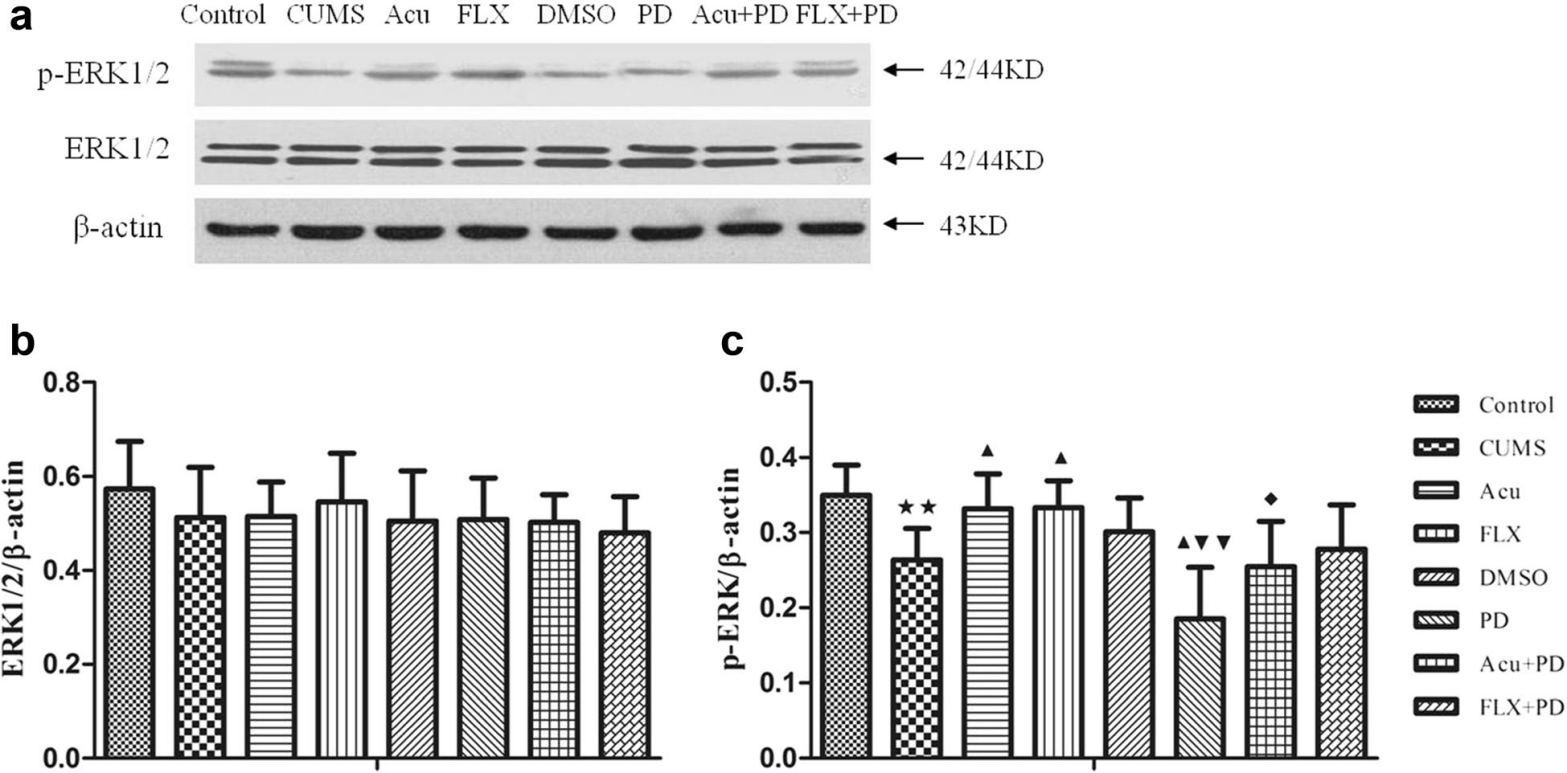
Western blot analysis of ERK1/2 and p-ERK1/2. **a** The representative immunoblot made from hippocampal tissue of rats. **b** The quantification of ERK1/2/β-actin ratio levels. **c** The quantification of p-ERK1/2/β-actin ratio levels. ^★★^ *P* < 0.01 vs. control group; ^▲^ *P* < 0.05 vs. CUMS group; ^▼▼^ *P* < 0.01 vs. DMSO group; ^◆^ *P* < 0.05 vs. Acu group. (mean ± SD, *n* = 6)

There was no significant difference in ERK1/2 protein expression in the hippocampus [*F*_(7,40)_ = 0.598, *P* > 0.05] among the groups; however, p-ERK1/2 expression different significantly among the groups [*F*_(7,40)_ = 6.804, *P* < 0.01] (Fig. 6 and Table 3). The p-ERK1/2 levels were down-regulated in the CUMS group (*P* < 0.01) compared to that in the control group, and acupuncture or fluoxetine treatment markedly increased these levels (*P* < 0.05 for both). The expression of p-ERK1/2 decreased more in the PD group than that in the CUMS group (*P* < 0.05) and the DMSO group (*P* < 0.01); however, there was no difference between the CUMS and DMSO groups (*P* > 0.05). The expression of p-ERK1/2 in the Acu + PD group was lower than that in the Acu group (*P* < 0.05). There was no significant difference in p-ERK1/2 between the FLX + PD and FLX groups, but the *P* value was close to the critical point (*P* = 0.067).

**Table 3:** Western blot analysis of ERK1/2 and p-ERK1/2

Group	ERK1/2/β-actin	p-ERK1/2/β-actin
Control	0.573 ± 0.101	0.350 ± 0.040
CUMS	0.512 ± 0.108	0.264 ± 0.041^★★^
Acu	0.515 ± 0.073	0.332 ± 0.046^▲^
FLX	0.546 ± 0.104	0.333 ± 0.036^▲^
DMSO	0.505 ± 0.107	0.301 ± 0.045
PD	0.508 ± 0.089	0.185 ± 0.069^▲▼▼^
Acu+PD	0.502 ± 0.059	0.255 ± 0.060^◆^
FLX+PD	0.480 ± 0.077	0.278 ± 0.059

^★★^ *P* < 0.01 vs. control group; ^▲^ *P* < 0.05 vs. CUMS group; ^▼▼^ *P* < 0.01 vs. DMSO group; ^◆^ *P* < 0.05 vs. Acu group. (mean ± SD, *n* = 6)

### Western blot analysis of CREB and p-CREB in the hippocampus

**Fig. 7:**
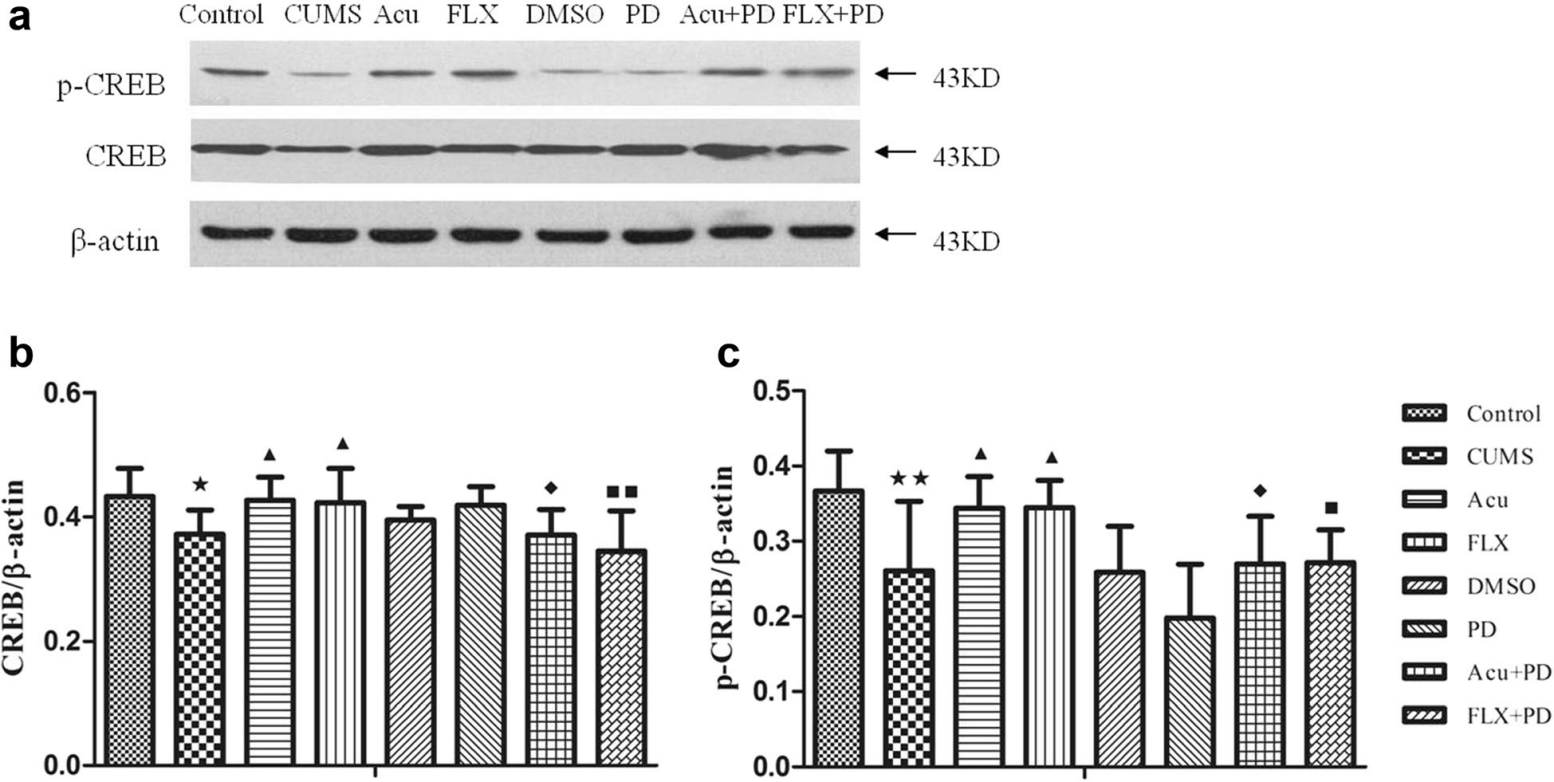
Western blot analysis of CREB and p-CREB. **a** The representative immunoblot made from hippocampal tissue of rats. **b** The quantification of CREB/β-actin ratio levels. **c** The quantification of p-CREB/β-actin ratio levels. ^★^ *P* < 0.05, ^★★^ *P* < 0.01 vs. control group; ^▲^ *P* < 0.05 vs. CUMS group; ^◆^ *P* < 0.05 vs. Acu group; ^■^ *P* < 0.05, ^■■^ *P* < 0.01 vs. FLX group. (mean ± SD, *n* = 6)

There was a significant difference in CREB [*F*_(7.40)_ = 3.323, *P* < 0.01] and p-CREB [*F*_(7,40)_ = 5.368, *P* < 0.01] expression among the groups (Fig. 7 and Table 4). The expression of both proteins was significantly down-regulated in the CUMS group (*P* < 0.05 and *P* < 0.01, respectively) compared to that in the control group. Acupuncture markedly increased CREB and p-CREB protein expressions (*P* < 0.05 for both). Fluoxetine also increased CREB and p-CREB protein expressions (*P* < 0.05 for both). There was no significant difference in the expression of CREB (*P* > 0.05) and p-CREB (*P* > 0.05) proteins among the CUMS, DMSO and PD groups; however, CREB and p-CREB protein expressions in the Acu + PD group were lower than those in the Acu group (*P* < 0.05 for both). Additionally, CREB and p-CREB protein expressions in the FLX + PD group were lower than those in the FLX group (*P* < 0.01 and *P* < 0.05, respectively).

**Table 4:** Western blot analysis of CREB and p-CREB

Group	CREB/β-actin	p-CREB/β-actin
Control	0.434 ± 0.045	0.367 ± 0.053
CUMS	0.371 ± 0.039^★^	0.261 ± 0.092^★★^
Acu	0.427 ± 0.037^▲^	0.344 ± 0.042^▲^
FLX	0.423 ± 0.055^▲^	0.345 ± 0.036^▲^
DMSO	0.394 ± 0.022	0.259 ± 0.061
PD	0.419 ± 0.030	0.198 ± 0.072
Acu+PD	0.371 ± 0.041^◆^	0.270 ± 0.063^◆^
FLX+PD	0.345 ± 0.065^■■^	0.272 ± 0.043^■^

^★^ *P* < 0.05, ^★★^ *P* < 0.01 vs. control group; ^▲^ *P* < 0.05 vs. CUMS group; ^◆^ *P* < 0.05 vs. Acu group; ^■^ *P* < 0.05, ^■■^ *P* < 0.01 vs. FLX group. (mean ± SD, *n* = 6)

### Western blot analysis of BDNF in the hippocampus

**Fig. 8:**
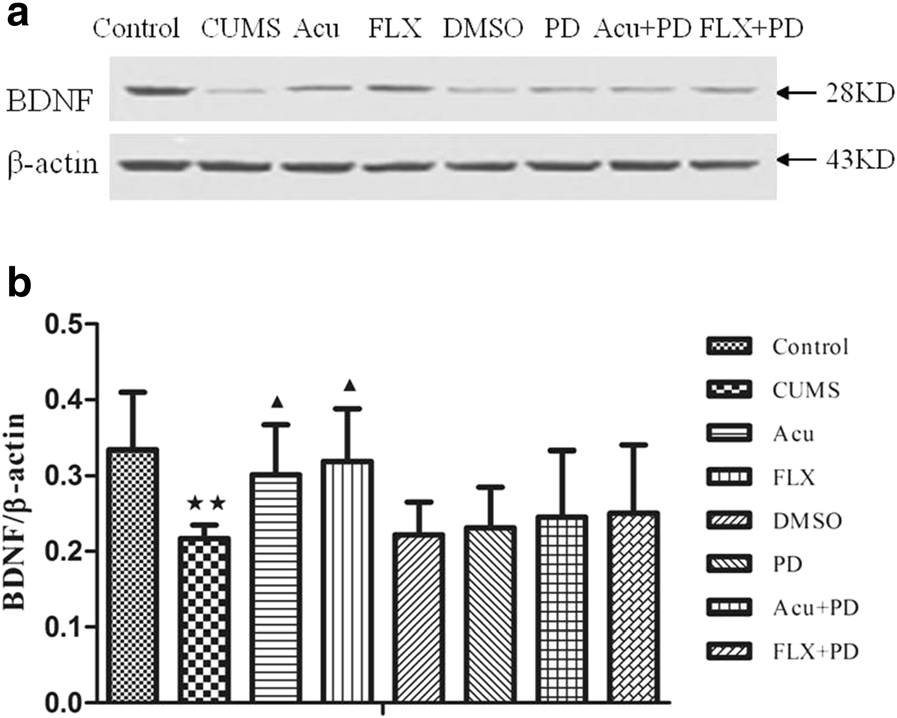
Western blot analysis of BDNF. **a** The representative immunoblot made from hippocampal tissue of rats. **b** The quantification of BDNF/β-actin ratio levels. ^★★^ *P* < 0.01 vs. control group; ^▲^ *P* < 0.05 vs. CUMS group. (mean ± SD, *n* = 6)

There was a significant difference in BDNF expression among the groups [*F*_(7.40)_ = 2.842, *P* < 0.05] (Fig. 8 and Table 5). It was significantly down-regulated in the CUMS group (*P* < 0.01) compared to that in the control group, and acupuncture or fluoxetine treatment markedly increased these levels (*P* < 0.05 for both). There was no significant difference in the expression of BDNF protein among the CUMS, CUMS and PD groups (*P* > 0.05). Additionally, the increase in BDNF protein induced by acupuncture or fluoxetine was not affected by PD98059 pretreatment (*P* > 0.05 for both).

**Table 5:** Western blot analysis of BDNF

Group	BDNF/β-actin
Control	0.334 ± 0.076
CUMS	0.217 ± 0.018^★★^
Acu	0.301 ± 0.066^▲^
FLX	0.319 ± 0.069^▲^
DMSO	0.222 ± 0.043
PD	0.231 ± 0.054
Acu+PD	0.245 ± 0.088
FLX+PD	0.250 ± 0.090

^★★^ *P* < 0.01 vs. control group; ^▲^ *P* < 0.05 vs. CUMS group. (mean ± SD, *n* = 6)

## Discussion

### Depression-like behaviors induced by CUMS

Chronic unpredictable mild stress (CUMS) can be used as a valid and reliable method by which to build an animal model of depression [35–37]. There are various methods to evaluate the model behaviors including the sucrose intake test (SIT), the open-field test (OFT) and the forced-swim test (FST). The decrease in sucrose consumption is considered as the inhibition of the brain reward system [38]. The OFT has been used widely to assess the anxiety behaviors [39]; however, it also can be used to evaluate the spontaneous activity in rodents [34, 40, 41]. Increased immobility in the FST is often anthropomorphized as an expression of despair; however, it can also be understood as a successful and adaptive behavioral response that functions to conserve energy [42]. Additionally, it was not recommended that the rats which received the surgery to perform the FST. As a result, we chose the SIT and OFT to analyze the depressive state of the model rats. The present study showed that CUMS induced an obvious decrease in sucrose intake and locomotion. The results suggested that CUMS decreased the sensitivity of model rats to sucrose and exploration, which were similar to the depressive symptoms, such as anhedonia and behavioral and cognitive dysfunction.

### Chronic unpredictable mild stress made the hippocampal extracellular signal-regulated kinase signaling pathway dysfunction

The ERK signaling pathway participates in neural proliferation, differentiation and neurogenesis and plays an important role in learning and memory [11, 43, 44]. A growing body of study demonstrates that the ERK signaling pathway is involved in the potential target for depression therapy [45–47], and inhibiting the ERK signaling pathway can block antidepressant medications activities [46, 48]. Furthermore, activated ERK protein can regulate transcription by controlling the phosphorylation of cyclic adenosine monophosphate response element binding protein (CREB) [12], which mediates the transcription of its target genes, such as BDNF mRNA and Bcl-2 mRNA. An early study found that the expression of CREB and p-CREB was decreased in the postmortem orbitofrontal cortex of patients with major depression disorder [49]. Another study found that CRE-DNA complexes, CREB protein, and CREB mRNA were reduced in the prefrontal cortex of depression patients who committed suicide [50]. Therefore, CREB also plays an important role in the physiology and pathology of depression [51] and treatment with antidepressants [52]. However, our early study showed that p-ERK1/2 and p-CREB, but not ERK1/2 and CREB, proteins decreased in the hippocampus and prefrontal cortex of rats exposed to CUMS [25]. Thus, in the present study, phosphorylated signaling proteins were tested in conjunction with their counterparts. Interesting, our finding showed that p-ERK1/2, CREB and p-CREB, but not ERK1/2, proteins reduced in the hippocampus of rats exposed to CUMS. It is known that only phosphorylated proteins exhibit full enzymatic activity; therefore, CUMS only reduced the expression of p-ERK1/2. Nevertheless, the depression-like behaviors were associated with the reductions of both CREB and p-CREB in the hippocampus.

### Effect of CUMS on expression of BDNF protein in the hippocampus

Previous studies have confirmed that hippocampal neurogenesis plays an important role in cognitive and emotional control [53] and that an increase in neural apoptosis can lead to mental disorders [54]. Additionally, a recent study gave direct evidence that the decreased neurogenesis was implicated in the pathogenesis of anxiety and depression [55]. Furthemore, research increasingly suggests that antidepressants can promote hippocampal neuron proliferation and differentiation [56–60]. BDNF is associated with neuroprotective and synaptic plasticity in the central nervous system [61], especially in the hippocampus [62, 63]. An early study proved that a reduction in BDNF in the hippocampus affects several behaviors related to depression [64]. Consistent with the previous studies, our results showed that the expression of BDNF protein decreased in rats exposed to CUMS, which demonstrated that the depression-like behaviors induced by CUMS were associated with BDNF protein in the hippocampus. Moreover, BDNF protein can activate the ERK signaling pathway by combining with its specific receptor tyrosine receptor kinase-B (Trk-B) [11]. Thus, reduction of BDNF protein might lead to a negative feedback to the ERK signaling pathway.

### Effect of PD98059 pretreatment on the behavior of CUMS rats and the ERK signaling pathway

Intracerebroventricular injection of PD98059, an inhibitor of ERK1/2, had no marked effect on the OFT and the expression of ERK1/2, CREB, p-CREB and BDNF in model rats; however, PD98059 pretreatment aggravated the decrease in the sucrose intake and the expression of p-ERK1/2 protein compared to those in the CUMS or DMSO group. The results were consistent with the property of PD98059 that prevents ERK1/2 from being phosphorylated without affecting the total protein, and suggested that inhibiting the expression of p-ERK1/2 protein in the hippocampus might lead to anhedonia. Additionally, there was no significant difference between the CUMS and DMSO groups in the SIT and OFT as well as the expressions of above proteins, which eliminated the disturbance of the surgery and DMSO.

### Acupuncture ameliorated depression-like behaviors and activated the ERK signaling pathway in the hippocampus

Acupuncture has been extensively used to treat depression in East Asian countries and has exhibited effective results in clinics. According to traditional Chinese medicine, Baihui (GV-20) and Yintang (GV-29) are points pertaining to Governor Meridian [34] and Governor Meridian has a direct contact with brain through channels and collaterals [41]. Thus, the acupoints of Baihui (GV-20) and Yintang (GV-29) are commonly used for treating depression [65]. In early studies, stimulation at Baihui (GV-20) and Yintang (GV-29) with electro-acupuncture (EA) exhibited antidepressant-like efficacy on in the SIT and OFT [34, 41]. In the present study, manual acupuncture performed at these acupoints also increased sucrose intake in the SIT and numbers of crossing and rearing in the OFT compared to the model rats, which proved that acupoint specificity can also play a significant role, independent of the electrical stimulus [25, 66, 67]. However, acupuncture was not as effective as fluoxetine in increasing sucrose intake in our study. In animals, the administration of acupuncture treatment is also a stimulation that is not as controllable as intragastric injection administration of a drug. Although the acupuncture has no effect on rats having a normal physiological state [25], the stimulation might influence the antidepressant-like effects of acupuncture on CUMS rats.

In the last few years, some studies indicated that the ERK signaling pathway was implicated in the antidepressant-like effects of acupuncture (manual acupuncture or EA). We previous found that manual acupuncture stimulation at Baihui (GV-20) and Neiguan (PC-6) increased the ratio of p-ERK1/2 to ERK1/2 and the ratio of p-CREB to CREB in the hippocampus and prefrontal cortex in CUMS rats [25]. In addition, Liu et al. showed that EA stimulation at Baihui (GV-20) and Yang-ling-quan (GB-34) mitigated depressive-like behaviors through increasing the p-ERK1/2 level in the hippocampus [26]. One research also found that EA stimulation at Baihui (GV-20) and Yintang (GV-29) acted on depression by enhancing the p-ERK1/2 in the hippocampus [68]. In the present study, we found that manual acupuncture performed at acupoints of Baihui (GV-20) and Yintang (GV-29) increased levels of p-ERK1/2, CREB, and p-CREB in the hippocampus. Pretreatment with PD98059 inhibited these improvements as well as the increase in sucrose intake and numbers of crossing and rearing. These results provided the direct evidence that the ERK signaling pathway was involved in the antidepressant-like effects of acupuncture.

### Effect of acupuncture on the expression of BDNF protein in the hippocampus

Recent research on depression focuses on neuroprotection, and an increasing number of studies support the notion that acupuncture is a rescuer of impaired neurogenesis [69]. It has been reported that acupuncture acts on depression by enhancing neuropeptide Y (NYP) in the hypothalamus [70] and amplifying neural progenitors (ANPs) proliferation, as well as preserving quiescent neural progenitors (QNPs) from apoptosis [71]. In our study, acupuncture markedly increased BDNF protein level, which provided further evidence to support the positive results. An early study found that inhibiting ERK1/2 phosphorylation can block the antidepressant-like consequences produced by infusing BDNF into the dentate gyrus [72]. Thus, we speculated that the ERK signaling pathway was implicated in acupuncture’s regulation of BDNF protein; however, the results showed that pretreatment with PD98059 did not abolish the effect of acupuncture on the BDNF protein. It is well known that CREB is an intersection of several signaling pathways. In addition to the ERK signaling pathway, cAMP-PKA and Ca^2+^/CaMK can also phosphorylate CREB and promote the transcription of BDNF mRNA. So an inhibited ERK signaling pathway can not suppress acupuncture on the regulation of BDNF protein. In addition, acupuncture’s neuroprotective effects may associate with the enhancement of p-p38 [68] and inhibition of pro-inflammatory cytokines [34, 73] and NF-kB signaling pathway [74] in the hippocampus. Moreover, in our previous study, it was found that acupuncture decreased the protein expressions of phosphor-Jun N-terminal kinase (p-JNK) and c-Jun, two important proteins in the JNK signaling pathway, and inhibited neural apoptosis mediated by caspases (data not published). Thus, the ERK signaling pathway is involved in the effects of acupuncture on neurotrophy and neurogenesis. However, the neuroprotective effect of acupuncture is not limited to the ERK signaling pathway.

## Conclusions

In conclusion, the present study demonstrated that depression-like behaviors of rats induced by CUMS were associated with the dysfunction of the ERK signaling pathway in the hippocampus. Importantly, our findings indicated that acupuncture treatment effectively mitigated depressive behaviors and the ERK signaling pathway was involved in the antidepressant-like effects of this treatment.

## Abbreviations

Acu: Acupuncture
BDNF: Brain-derived neurotrophic factor
CREB: cAMP response element-binding protein
CUMS: Chronic unpredictable mild stress
DMSO: Dimethylsulfoxide
EA: Elecro-acupuncture
ERK: Extracellular signal-regulated kinase
FLX: Fluoxetine
OFT: Open-field test
PD: PD98059
SIT: Sucrose intake test
Trk-B: Tyrosine receptor kinase-B
